# Impact of Inhaled Nitric Oxide (iNO) on the Outcome of COVID-19 Associated ARDS

**DOI:** 10.3390/jcm13195981

**Published:** 2024-10-08

**Authors:** Sandra Emily Stoll, Bernd W. Böttiger, Fabian Dusse, Nicolas Leister, Tobias Leupold, Christoph Menzel, Remco Overbeek, Alexander Mathes

**Affiliations:** 1 Department of Anesthesiology and Intensive Care Medicine, Faculty of Medicine and University Hospital Cologne, University of Cologne, 50937 Cologne, Germany; bernd.boettiger@uk-koeln.de (B.W.B.); fabian.dusse@uk-koeln.de (F.D.); nicolas.leister@uk-koeln.de (N.L.); tobias.leupold@uk-koeln.de (T.L.); christoph.menzel@uk-koeln.de (C.M.); remco.overbeek@uk-koeln.de (R.O.); alexander.mathes@uk-koeln.de (A.M.); 2 Department of Anesthesiology, Montefiore Medical Center, Albert Einstein College of Medicine, Bronx, NY 10467, USA

**Keywords:** COVID-19 associated ARDS, ARDS, inhaled nitric oxide, NO, iNO

## Abstract

**Background:** Inhaled nitric oxide (iNO) can improve oxygenation in acute respiratory syndrome (ARDS), has anti-inflammatory and antithrombotic effects, and can inhibit coronavirus- replication. The study aim was to investigate the impact of iNO in COVID-19 associated ARDS (CARDS) on oxygenation, the length of mechanical ventilation (MV), the level of inflammatory markers and the rate of thrombotic events during ICU stay. **Methods:** This was a retrospective, observational, monocentric study analyzing the effect of INO (15 parts per million) vs. non-iNO in adult ventilated CARDS patients on oxygenation, the level of inflammatory markers, and the rate of thrombotic events during ICU stay. Within the iNO group, the impact on gas exchange was assessed by comparing arterial blood gas results obtained at different time points. **Results:** Overall, 19/56 patients were treated with iNO, with no difference regarding sex, age, body mass index, and SOFA-/APACHE II- score between the iNO and non-iNO groups. iNO improved oxygenation in iNO-responders (7/19) and had no impact on inflammatory markers or the rate of thrombotic events but was associated with an increased MV length. **Conclusions:** iNO was able to improve oxygenation in CARDS in iNO-responders but did not show an impact on inflammatory markers or the rate of thrombotic events, while it was associated with an increased MV length.

## 1. Introduction

Despite the fact that COVID-19 associated acute respiratory syndrome (ARDS), so called “CARDS”, meets the Berlin definition for ARDS, CARDS presents with distinct differences compared to other forms of ARDS [1]. Patients with CARDS often show severe hypoxemia despite the maintenance of respiratory mechanics, which varies from other forms of ARDS [2]. Depending on the stage of CARDS, patients present differently: In the early phase, patients suffer from the “L-type” or “non-ARDS type” of CARDS, which is characterized by a low lung elastance and vasoplegic pulmonary vessels refractory to inhaled nitric oxide (iNO) [2]. In the late phase, patients show the “H-type” of CARDS, which presents with a high lung elastance. The late H-type, the classic ARDS type, is mainly characterized by pulmonary hypertension due to inflammation of the lung [2]. Moreover, patients suffering from CARDS present with an increased risk of pulmovascular micro- and macrothrombi due to endothelial injury and the activation of coagulation caused by SARS-CoV-2 [1,3]. Progressive hypoxia in CARDS induces pulmonary arterial vasoconstriction, further increasing pulmonary vascular resistance (PVR) and consequently decreasing right ventricular function. Unfortunately, hypoxemia in CARDS caused by shunting can only be partially reversed by additional supplemental oxygen [4]. Therefore, the mortality rate of CARDS remains as high as 40% [5].

iNO as a selective pulmonary vaso- and bronchodilator has been previously used in ARDS of another pathogenesis in the past to improve oxygenation by reducing the shunt fraction and ventilation/perfusion mismatch, and by reducing pulmovascular resistance in case of pulmonary hypertension [6,7,8,9,10]. The major advantages of iNO are its fast availability, its rapid inactivation in the bloodstream, the relatively low costs depending on the country, and few side effects with an appropriate dosage. Moreover, iNO is believed to reduce pro-inflammatory processes in CARDS [11]. The antimicrobial activity and viricidal effect of iNO can be explained by the suppression of the viral replication of SARS-CoV-2, particularly in the early stages of the disease [12,13,14,15,16,17,18]. The inhibition of the procoagulatory effects of SARS-CoV-2 by iNO is thought to potentially reduce thrombotic complications, which can be fatal in CARDS [19,20,21].

So far, little is known about the role of iNO in the clinical management of CARDS.

Therefore, this study aimed to compare the outcomes between patients with moderate or severe CARDS who were treated with iNO and patients suffering from CARDS who were treated without iNO. The study hypothesis was that iNO in the management of CARDS can improve the outcome parameters, such as the oxygenation, the length of ICU and hospital stay, the length of mechanical ventilation (MV), the 30-day mortality, the course of inflammatory markers, and the rate of thrombotic complications compared to patients treated without iNO.

## 2. Methods

### 2.1. Study Design

This CARDS study was performed as a retrospective, observational, monocentric cohort study focusing on COVID-19-positive adult patients who were admitted to the ICU of a quaternary care hospital in Germany for the management of CARDS between March 2020 and October 2021. CARDS was defined as ARDS according to the Berlin definition [22], with a confirmed COVID-19-positive reverse transcriptase PCR result.

### 2.2. Patients

All patients included in this study had moderate or severe CARDS (as per the Berlin definition) with a PaO_2_/FiO_2_ ratio ≤ 200 mmHg and received mechanical ventilation (MV). All patients were managed with lung-protective MV according to the ARDSnet trial’s recommendations [23], proning [24] (if PaO_2_/FiO_2_ ratio was ≤150 mmHg), and the administration of intravenous dexamethasone if required as per the Recovery trial [25] and according to the hospital’s protocol. Patients who received iNO for right heart failure and/or pulmonary hypertension and those who required higher doses of iNO were excluded from this study. All patients who received iNO underwent transthoracic echocardiography according to the hospital’s protocol before the initiation of iNO to assess the signs of pulmonary hypertension.

### 2.3. Application of iNO

INO was initiated and terminated according to the decision of the treating physician (if the PaO_2_/FiO_2_ ratio was <200 mmHg) as a rescue therapy for refractory hypoxemia despite the optimization of treatment options, and was continuously administered at 15 parts per million (ppm) as per the hospital’s protocol and according to the European expert recommendation [26,27]. This could vary quickly due to changes in minute ventilation or maneuvers such as suctioning or the transport of the patient for diagnostic or therapeutic reasons. Moreover, the dose of iNO was sequentially reduced (stepwise reduction of 5 ppm over 3 h) during the weaning phase of iNO, as per the decision of the treating physician. Positive responsiveness to iNO in our study was retrospectively defined by the study team as a cut-off value of the increase in PaO_2_/FiO_2_ ratio ≥ 20% within 6 h of iNO application [28]. The treating physicians did not use this cutoff value of responsiveness during the actual treatment phase; therefore, iNO was not necessarily stopped in case of non-responsiveness after 6 h. One reason for the termination of iNO was that the treatment was deemed clinically “effective and no longer required” or “ineffective and frustraneous” by the intensivist. Another reason for the termination of iNO was death or toxicity.

### 2.4. Ventilator Settings

All patients included in this study were ventilated with C6 Hamilton ventilators (Hamilton Medical AG, Bonaduz Swiss, Switzerland) in BIPAP mode (DUOPAP), a bilevel positive airway pressure ventilation mode, during their time on mechanical ventilation in the ICU. Moreover, all patients received lung-protective ventilator settings as per ARDS network recommendations—A tidal volume of ≤6 mL/kg predicted body weight (PBW), a plateau pressure ≤ 30 mbar with PEEP settings as per ARDS net table, and permissive hypercapnia (pH > 7.2) [23].

### 2.5. Data Retrieval

Demographic data and laboratory findings were retrospectively retrieved from electronic patient charts. The effects of iNO implementation on the oxygenation and decarboxylation were retrospectively assessed for direct effects by comparing arterial blood gas (ABG) results (cumulative mean value of the last three consecutive ABG results) immediately before the first iNO application with an ABG obtained 6 h after the first iNO administration. Additionally, the prolonged long-term effects of iNO were assessed by comparing the ABG results (cumulative mean values of the last three consecutive ABG results) taken before iNO administration with the ABG results (cumulative mean values of the last three consecutive ABG results) just before the end of iNO administration. Moreover, to assess the effect of iNO on inflammation, inflammatory markers such as C-reactive protein (CRP), procalcitonin, neutrophil/lymphocyte ratio and interleucin-6 (IL-6) were measured over a period of 10 days in the iNO versus the non-iNO group. Additionally, the length of MV, length of hospital and ICU stay, 30-day mortality, rate of thrombotic events, and D-dimer levels during the entire ICU stay were assessed in the iNO versus the non-iNO group.

### 2.6. Ethical Approval

This study was approved by the Ethics Committee of the University of Cologne, Germany (21-1553-retro 14 December 2021) as a low-risk study with a waiver of individual patient informed consent due to the retrospective nature and the performance of an analysis of pseudonymized data. Informed consent was not obtained from each patient. This study was conducted in accordance with the Declaration of Helsinki.

### 2.7. Sample Size

Owing to the retrospective nature of this study, only the assessment of a convenience sample of all patients fulfilling the inclusion criteria was possible.

### 2.8. Statistical Analysis

In this study, continuous variables were compared using means with standard deviation (SD) and medians with interquartile ranges (IQR), according to data type and distribution. Categorical variables were compared by using frequency counts and percentages. For the comparison of all parameters between the groups, independent Student’s *t*-test or Wilcoxon signed-rank test, as appropriate for continuous variables, and chi-square test with Yates correction for categorical variables were used. For comparisons of all parameters before and after iNO application, paired-samples Student’s *t*-test or Wilcoxon signed-rank test were used as appropriate for continuous variables, and the chi-square test with Yates correction for categorical variables was used. A two-sided *p*-value ≤ 0.05 was chosen to indicate statistical significance. Statistical analyses were performed using SPSS Statistics version 29 (IBM, Armonk, NY, USA).

## 3. Results

### 3.1. Patients’ Demographics and Characteristics in the iNO vs. the Non-iNO Group

This retrospective study included 56 patients who received ICU treatment between March 2020 and October 2021. Nineteen of fifty-six (33.93%) patients underwent a treatment trial with iNO (see Table 1). According to the patients’ demographics, the iNO group and the non-iNO group presented a similar patient cohort regarding the distribution of sex, mean age, variables of gas exchange in the arterial blood gas on admission, and mean SOFA and APACHE II scores on admission (see Table 1). In the iNO group, 17/19 (89.47%) patients vs. 32/37 (86.49%) in the noniNO group underwent a treatment trial with dexamethasone (6 mg iv. once daily for 10 days). The differences between both groups were as follows: Proning as a treatment trial to improve oxygenation was performed in 94.74% of patients in the iNO group vs. only 54.05% in the non-iNO group. Furthermore, patients in the iNO group were more likely to have a wild- or alpha-variant of COVID, whereas patients in the non-iNO group presented with the wild-, alpha-, and beta-variants of COVID (see Table 1).

### 3.2. Patients’ Demographics and Characteristics in the iNO-Responder vs. Nonresponder Group

Positive responsiveness to iNO was defined after data acquisition as an increase in the PaO_2_/FiO_2_ ratio by ≥20% within 6 h of iNO application. Of the 19 patients receiving iNO, 7 (36.84%) were responders to iNO and 12 were nonresponders (63.16%) according to this definition (Table 2). There was no significant difference in iNO-responders vs. nonresponders regarding sex (6/7 male in the iNO responder vs. 7/12 in the iNO-nonresponder group, *p* = 0.33), age (54.00 ± 18.95 years vs. 63.78 ± 12.34 years, *p* = 0.19), BMI (33.68 ± 10.03 kg/m^2^ vs. 31.51 ± 4.98 kg/m^2^, *p* = 0.53), APACHE II (26.43 ± 14.94 vs. 23.42 ± 12.40, *p* = 0.66) or SOFA score (8.57 ± 4.76 vs. 7.58 ± 3.58, *p* = 0.61), as seen in Table S1 in the supplement.

### 3.3. ABG Results before and after iNO

All patients in the iNO group in this study received iNO at a dose of approximately 15 ppm. This could vary due to changes in minute ventilation or maneuvers, such as suctioning or transport of the patient for diagnostic or therapeutic reasons. The mean start date of treatment with iNO was 1.98 ± 4.96 days after admission to ICU, and the mean duration of iNO was 103.53 ± 92.99 h (median 67.50 h; IQR: 38.00–111.00 h).

### 3.4. ABG Results before vs. 6 h after iNO Initiation in iNO-Responders versus -Nonresponders

Out of 19 patients in the iNO group, 7 were responders to iNO with a significant improvement in SpO_2_ and PaO_2_/FiO_2_ ratio, as well as a reduction in the estimated shunt and FiO_2_ requirements (see Table 2) within the first 6 h of iNO application. Of the 19 patients presented as iNO-nonresponders, 12 showed no change, or even a decline, in oxygenation and the progress of the estimated shunt under iNO (see Table 2).

#### 3.4.1. ABG Results Pre-iNO and before Termination of iNO

Comparing the cumulated ABG results (mean values of the last three consecutive ABGs) before the initiation of iNO compared to the ABG results before termination of the iNO therapy, we see that there was a significant difference between ABG results before iNO initiation and before iNO termination—the FiO_2_ requirements (0.52 ± 0.17 vs. 0.66 ± 0.17; *p* = 0.01) and shunt volume (18.20 ± 6.51 vs. 23.74 ± 4.52%; *p* = 0.01) in the ABG were reduced at the end of the therapy with iNO compared to the values before iNO initiation (see Table 3). In contrast, SpO_2_ (95.36 ± 2.19 vs. 94.23 ± 1.58%; *p* = 0.05) and PaO_2_/FiO_2_ ratio (183.97 ± 49.96 vs. 138.80 ± 32.50 mmHg; *p* = 0.01) were significantly increased before iNO termination compared to values before iNO initiation (see Table 3). There were no significant differences in paO_2_, pCO_2_ and pH values measured before iNO initiation compared with the values before the termination of iNO (see Table 3).

#### 3.4.2. Impact of iNO vs. Non-iNO on Oxygenation (PaO_2_/FiO_2_ (mmHg))

**Figure 1:**
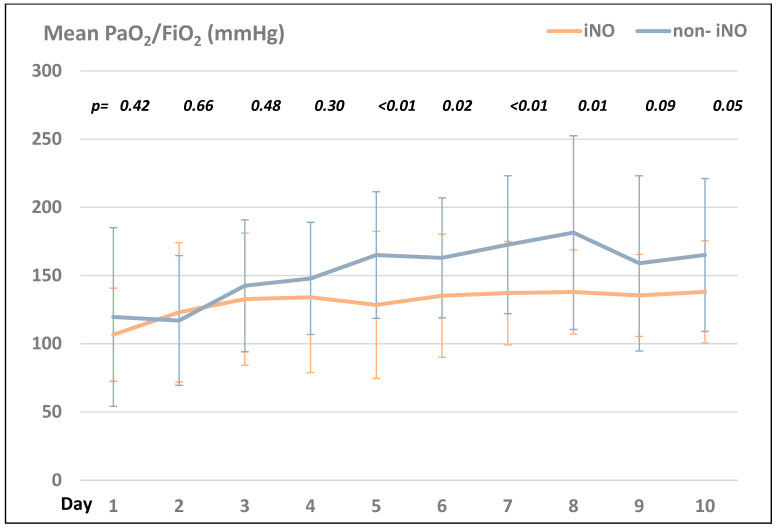
Comparison of the mean PaO_2_/FiO_2_ (mmHg) in the iNO- vs. the non-iNO group within the first 10 days after ICU admission.

Comparing the PaO_2_/FIO_2_ ratio in the iNO- and the non-iNO groups, the non-iNO group presented with higher ratios compared to the iNO group during the first ten days after ICU admission (see Figure 1).

### 3.5. Ventilator Settings in the iNO vs. Non-iNO Group

All patients included in this study were ventilated with C6 Hamilton ventilators (Hamilton Medical AG, Bonaduz Swiss, Switzerland) in BIPAP-mode (DUOPAP), a bilevel positive airway pressure ventilation mode, during their ICU stay. Moreover, all patients received lung-protective ventilator settings, as per the ARDS network recommendations, with a tidal volume of ≤6 mL/kg predicted body weight (PBW), a plateau pressure ≤ 30 mbar with PEEP settings as per the ARDS net table, and permissive hypercapnia (pH > 7.2) [23].

**Figure 2:**
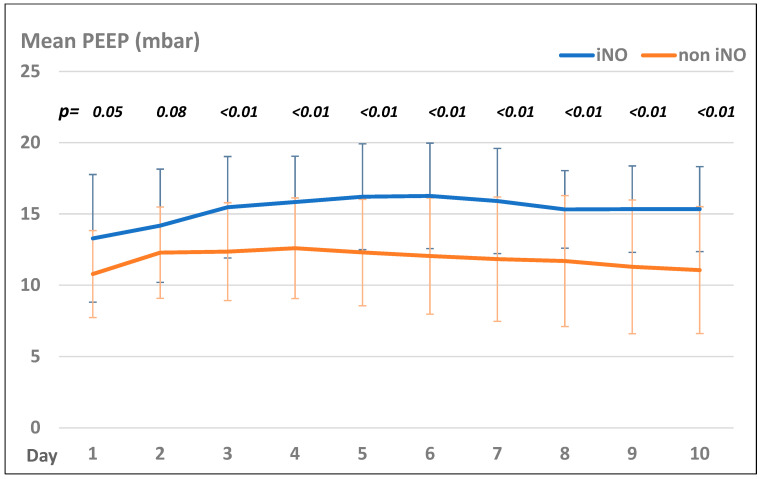
Mean PEEP levels (mbar) in the iNO and non-iNO group.

Patients in the iNO group presented with significantly higher PEEP values than those in the non-iNO group, but both groups showed the same trend of PEEP settings during their treatment course (see Figure 2).

### 3.6. Trend of Inflammatory Markers in the iNO and Non-iNO Group

**Figure 3:**
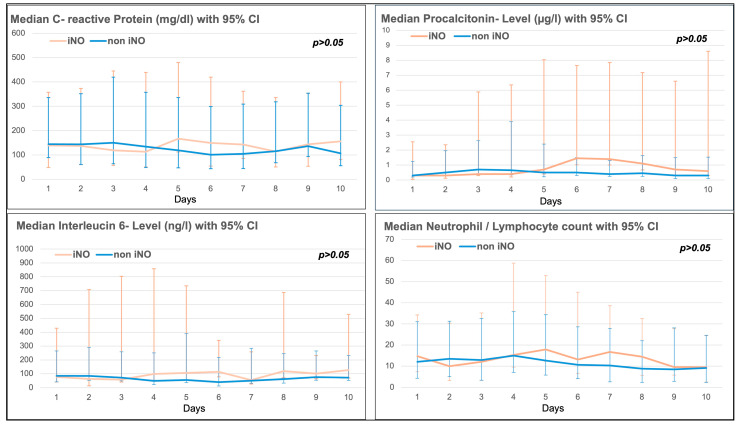
Inflammatory markers (C-reactive protein (CRP), Procalcitonin, Interleucin-6 and Neutrophil/Lymphocyte ratio) in the iNO vs. non-iNO group.

There was no significant difference in the 10-day trend of inflammatory markers such as C-reactive protein (CRP), procalcitonin, Interleucin-6 (IL-6), or the neutrophil/lymphocyte ratio between the iNO and non-iNO groups (see Figure 2). Inflammatory markers seemed to be higher in the iNO group between day 4 and day 10 after ICU admission (see Figure 3), with a mean start time of iNO of 1.98 ± 4.96 days after admission to ICU, and a median duration of iNO of 67.50 h (IQR 38–111 h).

### 3.7. Clinical Outcome Parameters iNO vs. Non-iNO Group

#### 3.7.1. Length of Mechanical Ventilation (MV) and ICU and Hospital Stay

The length of MV in patients with iNO vs. non-iNO as well as the length of ICU stay increased in patients who received iNO. In contrast, there was no difference in the length of hospital stay (see Table 4) between iNO and non-iNO.

#### 3.7.2. 30-Day Mortality

The 30-day mortality rate was higher in patients who received iNO than in non-iNO patients (see Table 4).

#### 3.7.3. Renal Replacement Therapy

Two of 19 patients treated with iNO versus one of 37 patients treated without iNO required renal replacement therapy (*p* = 0.26) during their ICU stay (Table 4).

#### 3.7.4. Thrombotic Events in the iNO versus Non-iNO Group

There was no significant difference in documented thrombotic events between the iNO group (26.31%) and the non-iNO group (10.81%, *p* = 0.14, see Table 4). Furthermore, there was no significant difference in D-dimer levels during the first 10 days after ICU admission (Table 5).

#### 3.7.5. Outcome Parameters in the iNO-Responder vs. -Nonresponder Group

There was no significant difference in iNO-responders compared to nonresponders regarding length of ICU (26.37 ± 16.41 days vs. 20.82 ± 16.98 days, *p* = 0.5) or hospital stay (27.23 ± 16.33 days vs. 22.96 ± 18.97, *p* = 0.63) and length of mechanical ventilation (23.61 ± 17.41 days vs. 17.65 ± 12.47, *p* = 0.40), thrombotic complications (2/7 in the iNO-responder vs. 3/12 in the iNO-nonresponder group, *p* = 0.87), renal replacement therapy (4/7 in the iNO-responder vs. 3/12 in the iNO-nonresponder group, *p* = 0.16) and 30-day mortality (6/7 in the iNO-responder vs. 10/12 in the iNO-nonresponder group, *p* = 0.89).

#### 3.7.6. Signs of iNO Toxicity

None of the patients in our study treated with iNO presented with methemoglobinemia or elevated NO_2_.

## 4. Discussion

In our study, iNO did not affect inflammatory markers or the rate of thrombotic events in patients with CARDS. Moreover, our study revealed that iNO improved oxygenation (PaO_2_/FiO_2_ ratio) by reducing the estimated shunt in iNO-responders within 6 h of iNO initiation, but not in iNO-nonresponders. In contrast, the administration of iNO was associated with an increased length of MV and an increased length of ICU stay, but not any change in hospital stay, and it was associated with an increased 30-day mortality.

Our results are consistent with those of previous studies. A Cochrane meta-analysis of 14 randomized controlled trials could be used to detect an association between iNO and the improvement of oxygenation (improvement of PaO_2_/FiO_2_ ratio) with the use of iNO but could not reveal a positive impact on 30-day mortality [1,7,29,30]. Additionally, in our study, the use of iNO was associated with an increased mortality, a reduced number of ventilator-free days, and an elevated length of ICU stay. The reason for this might be that patients in this group potentially presented with a more severe progression of CARDS, and therefore were chosen to receive iNO therapy. This is reflected in the higher number of patients in the proning position and the higher PaO_2_/FiO_2_ ratios throughout the first ten days after ICU admission in the iNO group compared to the non-iNO group. Based on current evidence, the Surviving Sepsis campaign guidelines for managing patients with CARDS strongly advocate against the routine use of iNO in CARDS but recommend only the use of iNO as an adjunct “rescue therapy” in refractory hypoxemia if oxygenation can be improved by iNO [31]. Moreover, iNO should be discontinued if there is no positive effect within the first 24 h after its initiation [31,32]. A systematic review and meta-analysis by Alqahtani et al. showed a positive response rate regarding the improvement of oxygenation with iNO therapy in 66% of patients [33]. In our study, iNO only presented a positive response rate of approximately 37%. This might be explained by the different responsiveness to iNO at the different stages of CARDS. In our study, iNO was started relatively early, with a mean start time around two days after ICU admission, whereas other studies with a higher responsiveness rate of iNO started iNO in the later course of ICU admission. In the early stage of CARDS, the vasoplegic pulmonary vessels and a dysfunctional pulmonary vascular endothelium combined with micro- and macrothrombi may be refractory to iNO [1]. In contrast, there is some evidence that iNO is more effective in the early stages of the disease due to the suppression of the viral replication and the procoagulatory effects of the virus. As a limitation, it is unclear in our study at which stage of the disease our patients were admitted to the ICU, and our study group was too small to draw conclusions regarding this aspect. The comparison of studies regarding the use of iNO is difficult because previous studies vary in dosage, timing (early versus late initiation of iNO), duration, and outcome parameters. Currently, there are no predictive markers that indicate which patients may benefit from iNO therapy. Therefore, studies in the past came to different conclusions regarding whether patients with mild, moderate, or severe CARDS benefit from iNO. It remains unclear whether the benefit of iNO depends on the presence or the absence of pulmonary hypertension, or the presence of other demographic influences or comorbidities. There is a suspicion that comorbidities such as metabolic disorders, such as diabetes, reduce the capacity of hemoglobin to carry NO and impair the synthesis of NO, potentially resulting in endogenous NO depletion. This NO depletion in metabolic disorders may represent a risk factor for a poor outcome and may explain the inhomogeneous therapeutic effects of iNO in different patient groups [34]. The same applies to patients’ medications, such as ACE2-inhibitors, since the ACE2-receptor is known to upregulate NO synthesis. Moreover, the timing and dosage of an ideal iNO therapy are undefined, and there is no evidence yet whether iNO should be administered early as prophylaxis versus late, or even only as a rescue therapy for refractory hypoxemia. Currently, there is also uncertainty regarding the adequate dosage and duration of iNO—a low vs. high dose, an intermittent or continuous dosage, and a varied vs. a fixed dose. An adequate effect of iNO should be already detectable within 6 h after the initiation of iNO [28] (as chosen as the cut-off timeframe to assess the effectiveness of iNO in our study), or at the latest within the first 24 h, as in a study by Suleiman et al. [30]. There is also growing evidence that the sensitization to an iNO treatment occurs with iNO use after >96 h [1,35]. However, there is a lack of criteria for adequate dose adjustment, the duration of iNO, and standardized weaning criteria. After a treatment duration of more than five days, the therapeutic effect is expected to disappear owing to the downregulation of the endogenous NO synthesis.

A major disadvantage of iNO is that it must be closely monitored for toxicological effects, such as the production of methemoglobin (methemoglobin > 5%) and nitrogen dioxide (NO_2_ > 2 ppm). In our study, no patient had signs of methemoglobinemia or elevated NO_2-_ levels. Another side effect of iNO is the inhibition of platelet aggregation, which may play a beneficial role in CARDS, where microthrombi can occur in pulmonary vessels [19,20]. In our study, iNO neither reduced the rate of thrombotic events nor reduced the D-dimer levels compared to the non-iNO group. Additionally, iNO is known to have immunoregulatory effects and can reduce viral entry into the host cell via the ACE2-receptors. NO is released endogenously by the airway epithelium, and rapidly inactivated in the bloodstream [17]. In ICU patients with impaired NO release, the inhalation of NO might help to improve the immunoregulatory response and to reduce the virus replication at an early stage of the COVID disease [36,37]. Additionally, in CARDS, iNO is believed to reduce proinflammatory processes [11]. In contrast, iNO did not affect the inflammatory response in our study. This is in contrast to data published by other study groups, which found that IL-6 is a good predictor of responsiveness to iNO, since it was found that IL-6 levels are three times higher in patients who received iNO [38]. In our study, there were no significant differences in IL-6, CRP, procalcitonin levels or neutrophil/lymphocyte ratio in the iNO versus the non-iNO group. IL-6 was only non-significantly elevated in the iNO group between days four and ten after ICU admission.

Our study had several limitations. Given the retrospective, single-center nature of this study and due to the small sample size, including the small number of iNO-responders (only 37%), our study might lack generalizability. Moreover, the distribution of the COVID subvariants was unequally distributed among the iNO group (79% wild type) and the non-iNO group (81% beta variant), which could have influenced the outcome. Another limitation of this study was the lack of continuous pulmonary artery pressure monitoring to evaluate the effects of iNO on pulmonary pressure. Patients receiving iNO were only assessed for signs of pulmonary hypertension using transthoracic echocardiography before the initiation of iNO. The decision to use iNO was made by the treating physician, in all cases as a “rescue therapy” in refractory hypoxemia despite lung-protective ventilation, proning, and negative fluid balance. Therefore, the starting point of the iNO therapy was heterogeneous, and may have occurred at different stages of CARDS in our study group. Additionally, we cannot fully exclude that there was a treating bias, whereby patients with a more severe progress of CARDS received iNO resulting in a higher ICU length of stay, an elevated length of mechanical ventilation and a higher 30-day mortality compared to the non-iNO group.

Nevertheless, our study has several strengths. iNO was subsequently titrated to 15 ppm according to the hospital´s protocol for CARDS patients throughout the treatment phase (except the weaning phase), and the dosage was monitored by acoustic alarms that were responded to directly. Owing to the COVID pandemic, the study cohort and treatment of this study cohort were homogenous and standardized.

## 5. Conclusions

In patients with CARDS, the continuous application of 15 ppm iNO could improve oxygenation by reducing shunts in iNO responders. In contrast, the application of iNO did not have an impact on inflammation or the incidence of thrombotic events but was associated with a longer duration of MV.

## Figures and Tables

**Table 1:** Demographic data and ABG results of patients suffering from CARDS treated with iNO vs. non-iNO.

Variable	iNO-Group *n*= 19	Non-iNO Group *n* = 37	All Patients *n* = 56	*p*- Value
Patients’ Demographics and Characteristics				
Age (years)	60.18 ± 15.37	66.44 ± 11.81	64.32 ± 13.33	0.10
Sex (male)	13/19 (68.42%)	26/37 (70.27%)	39/56 (69.64)	0.56
Body mass index (kg/m^2^)	32.31 ± 7.06	29.66 ± 7.97	30.56 ± 7.71	0.23
SOFA-score on admission	7.95 ± 3.95	7.97 ± 4.48	7.96 ± 4.27	0.98
APACHE II Score on admission	24.53 ± 13.06	21.95 ± 8.83	22.82 ± 10.41	0.39
COVID variant				
Wild type	4/19 (21.05%)	22/37 (59.46%)	26/56 (46.43%)	<0.01
Alpha (United Kingdom)	15/19 (78.95%)	12/37 (32.43%)	27/56 (48.21%)	
Beta (South African)	-	3/37 (8.11%)	3/56 (5.36%)	
Highest IL-6 level at admission (ng/L)	77 (37.00–351.75)	84.50 (43.25–180.50)	81 (43.00–191.00)	0.12
Highest IL-6 level during ICU-stay (ng/L)	824.00 (240.75–10,852.50)	377.00 (124.75–835.00)	444.50 (142.00–1572.00)	0.50
Dexamethasone (6 mg OD iv. for 10 days)	17/19 (89.47%)	32/37 (86.49%)	49/56 (87.50%)	0.75
Proning	18/19 (94.74%)	20/37 (54.05%)	38/56 (67.86%)	<0.01
ABG-results				
Highest PaO_2_/FiO_2_ ratio on admission day (mmHg)	203.05 ± 92.85	183.04 ± 77.20	189.83 ± 82.55	0.40
Lowest PaO_2_/FiO_2_ ratio on admission day (mmHg)	106.61 ± 34.10	119.57 ± 65.47	115.18 ± 56.79	0.42
Highest PaO_2_/FiO_2_ ratio during ICU-stay (mmHg)	474.32 ± 208.37	507.16 ± 204.44	496.02 ± 204.49	0.57
Lowest PaO_2_/FiO_2_ ratio during ICU-stay (mmHg)	68.85 ± 17.02	80.18 ± 24.63	76.34 ± 22.83	0.79
Highest estimated shunt fraction (ABG) during ICU-stay (%)	41.25 ± 7.65	43.71 ± 36.82	42.87 ± 30.13	0.78
Mean estimated shunt fraction (ABG) during ICU-stay (%)	26.53 ± 5.18	25.54 ± 5.24	25.88 ± 5.20	0.51

ABG, arterial blood gas; APACHE II, Acute Physiology and Chronic Health Evaluation; ICU, Intensive Care Unit; IL-6, interleucin 6; OD, once daily; PaO_2_/FiO_2_ ratio, PaO_2_ (arterial partial pressure of oxygen)/FiO_2_ (fraction of inspired oxygen); SOFA, sepsis-related organ failure assessment score. Data are presented as mean ± standard deviation, median (interquartile range), or number (percent).

**Table 2:** ABG results in iNO-responders vs. -nonresponders before iNO initiation.

	iNO-Responders			iNO-Nonresponder		
Variable:	Cumulated Pre-iNO ABG (Mean of 3 Baseline ABGs) *n = 7*	ABG 6 h Post iNO *n = 7*	*p*-Value	Cumulated Pre-iNO ABG (Mean of 3 Baseline ABGs) *n = 12*	ABG 6 h Post iNO *n = 12*	*p*-Value
Cumulative FiO_2_	0.74 ± 0.19	0.58 ± 0.13	0.03	0.63 ± 0.16	0.59 ± 0.12	0.13
Cumulative SpO_2_ (%)	94.8 + 2.96	96.70 ± 2.40	0.05	94.86 ± 2.80	93.13 ± 2.14	0.03
Cumulative PaO_2_ (mmHg)	88.69 ± 27.27	117.94 ± 65.72	0.06	95.44 ± 25.86	72.27 ± 7.82	<0.01
Cumulative PaCO_2_ (mmHg)	51.16 ± 9.85	50.04 ± 9.70	0.39	64.76 ± 18.82	54.67 ± 15.36	0.05
Cumulative pH	7.35 ± 0.07	7.36 ± 0.09	0.38	7.29 ± 0.11	7.36 + 0.11	0.04
Cumulative PaO_2_/FiO_2_ ratio (mmHg)	127.19 + 47.52	207.71 ± 98.27	0.02	158.41 ± 46.93	126.30 ± 22.91	0.10
Cumulative estimated shunt (%)	25.76 ± 6.70	16.77 ± 7.10	<0.01	20.86 ± 5.56	24.51 ± 5.26	0.03

**Table 3:** ABG results before iNO-initiation (baseline) vs. before iNO termination.

Variable	“Baseline” Cumulated ABG before Initiation of iNO (Mean of 3 Consecutive ABGs) *n* = 19	“Endpoint” Cumulated ABG before Termination of iNO (Mean of 3 Consecutive ABGs) *n* = 19	*p*-Value
Cumulative FiO_2_	0.66 ± 0.17	0.52 ± 0.17	0.012
Cumulative SpO_2_ (%)	94.23 ± 1.58	95.36 ± 2.19	0.05
Cumulative PaO_2_ (mmHg)	86.41 ± 11.80	89.91 ± 16.62	0.47
Cumulative PaCO_2_ (mmHg)	59.19 ± 15.83	54.90 ± 8.67	0.29
Cumulative pH	7.32 ± 0.10	7.37 ± 0.08	0.09
Cumulative PaO_2_/FiO_2_ ratio (mmHg)	138.80 ± 32.50	183.97 ± 49.96	0.006
Cumulative estimated shunt (%)	23.74 ± 4.52	18.20 ± 6.51	0.009

**Table 4:** Outcome parameters in the iNO vs. non-iNO group.

Outcome Parameters	iNO *n* = 19	Non-iNO *n* = 37	All *n* = 56	*p*- Value
Length of Hospital stay (days)	24.53 ± 17.70	22.86 ± 20.06	23.43 ± 19.15	0.76
Length of ICU stay (days)	22.86 ± 16.54	15.49 ± 11.01	17.99 ± 13.47	0.051
Length of invasive ventilation (days)	19.85 ± 14.31	10.54 ± 9.66	13.69 ± 12.12	0.006
Renal replacement therapy	2/19 (10.53%)	1/37 (2.79%)	3/56 (5.36%)	0.26
Thrombotic complications	5/19 (26.31%)	4/37 (10.81%)	9/56 (16.07%)	0.14
30-day mortality	16/19 (84.21%)	15/37 (40.54%)	31/56 (55.36%)	0.002

ICU = Intensive Care Unit, *n* = number. Data are presented as mean ± standard deviation of as numbers (with percentage).

**Table 5:** 10-day trend of D-dimers in the iNO vs. non-iNO group.

Variable	iNO	Non-iNO	All	*p*-Value
D-Dimer	N = Number/Median (Interquartile Range) Mean ± SD			
Day 1	N = 11 2.09 ± 1.58 1.87 (0.75–3.12)	N = 29 7.50 ± 11.75 2.03 (1.08–6.76)	N = 40 6.01 ± 10.28 1.97 (0.78–5.38)	0.14
Day 2	N = 18 5.83 ± 10.83 1.96 (0.77–4.11)	N = 33 7.95 ± 12.30 1.86 (1.04–6.85)	N = 51 7.20 ± 11.74 1.97 (0.93–4.63)	0.27
Day 3	N = 16 5.49 ± 8.95 2.03 (0.77–4.11)	N = 33 8.68 ± 11.00 2.69 (1.25–14.22)	N = 49 7.64 ± 10.39 2.03 (1.25–11.15)	0.16
Day 4	N = 19 6.54 ± 10.49 1.99 (1.4–6.76)	N = 30 8.27 ± 10.75 2.14 (1.20–15.96)	N = 49 7.60 ± 10.57 1.99 (1.24–10.20)	0.29
Day 5	N = 19 6.13 ± 9.61 2.77 (1.24–6.14)	N = 30 5.99 ± 7.61 2.73 (1.18–9.01)	N = 49 6.04 ± 8.34 2.77 (1.23–7.06)	0.48
Day 6	N = 19 6.48 ± 9.28 2.89 (1.15–8.18)	N = 29 6.96 ± 10.11 2.33 (1.09–8.53)	N = 48 6.77 ± 9.69 2.73 (1.14–7.87)	0.43
Day 7	N = 17 5.79 ± 6.83 3.58 (1.73–7.20)	N = 28 7.68 ± 11.26 3.72 (1.16–6.54)	N = 45 6.97 ± 9.78 3.66 (1.4–6.71)	0.27
Day 8	N = 17 4.69 ± 4.55 2.55 (2.05–6.72)	N = 28 7.03 ± 9.75 3.33 (1.28–6.93)	N = 45 6.14 ± 8.19 2.60 (1.57–6.82)	0.18
Day 9	N = 15 7.18 ± 9.15 4.36 (2.10–8.54)	N = 26 7.54 ± 8.45 4.15 (1.94–9.01)	N = 41 7.41 ± 8.60 4.16 (2.06–8.21)	0.45
Day 10	N = 16 7.34 ± 8.60 4.99 (1.74–10.77)	N = 26 7.88 ± 10.05 4.04 (1.74–7.24)	N = 42 7.67 ± 9.42 4.19 (1.77–9.16)	0.43

## Data Availability

The data that support the findings of this study are not openly available owing to reasons of sensitivity and are available from the corresponding author upon reasonable request. Data were stored as controlled-access data at the University Hospital Cologne, Germany.

## Supplementary Materials

The following supporting information can be downloaded at: https://www.mdpi.com/article/10.3390/jcm13195981/s1, Table S1: Demographic differences in iNO responders and nonresponders.
