# Supplementary material for: Impact of Inhaled Nitric Oxide (iNO) on the Outcome of COVID-19 Associated ARDS
Source: J Clin Med. 2024 Oct 8;13(19):5981. doi: 10.3390/jcm13195981 (PMC11478273; doi:10.3390/jcm13195981)
Supplement: Supplementary file 1 [file jcm-13-05981-s001.zip › jcm-3189074-supplementary.pdf]

Table S1 Demographic differences in iNO responders and nonresponders.

Variable	iNO-Group All N = 19	iNO Responder N = 7	iNO Nonresponder N = 12	*p*-Value
**Patients' Demographics and Characteristics**				
Age (years)	60.18 ± 15.37	54.00 ± 18.95	63.78 ± 12.34	0.19
Sex (male)	13/19 (68.42%)	6/7 (85.71%)	7/12 (58.33%)	0.33
Body mass index (kg/m^2)	32.31 ± 7.06	33.68 ± 10.02	31.51 ± 4.98	0.53
SOFA-Score on admission	7.95 ± 3.95	8.57 ± 4.76	7.58 ± 3.58	0.61
APACHE II Score on admission	24.53 ± 13.06	26.43 ± 14.94	23.42 ± 12.40	0.64
COVID variant<br>Wild type<br>Alpha (United Kingdom)<br>Beta (South African)	4/19 (21.05%)<br>15/19 (78.95%)<br>-	2/7 (28.57%)<br>5/7 (71.43%)	2/12 (16.67%)<br>10/12 (83.33%)	0.60
Highest IL-6 levels on admission day (ng/l)	77 (37.00–351.75)	66.00 (48.25–46,293.00)	82.50 (28.00–223.25)	0.16
Dexamethasone (6 mg OD iv. for 10 days)	17/19 (89.47%)	7/7 (100%)	10/12 (83.33%)	0.51
Proning	18/19 (94.74%)	6/7 (85.71%)	12/12 (100%)	0.37
ABG-results				
Highest PaO_2/FiO_2 ratio on admission day (mmHg)	203.05 ± 92.85	220.42 ± 135.09	192.92 ± 62.05	0.55
Highest shunt fraction on admission day	29.31 ± 7.32	31.06 ± 5.09	28.58 ± 8.16	0.54

ABG, arterial blood gas; APACHE 2, Acute Physiology and Chronic Health Evaluation, ICU = Intensive Care Unit, IL-6 = interleucin 6, OD once daily; PaO_2/FiO_2 ratio, PaO_2 (arterial partial pressure of oxygen)/FiO_2 (fraction of inspired oxygen), SOFA = Sepsis-related organ failure assessment score, UKK = United Kingdom. Data are presented as mean ± standard deviation, median (interquartile range), or number (percent).
